# Rapid Vector-Based Peak Fitting and Resolution Enhancement for Correlation Analyses of Raman Hyperspectra

**DOI:** 10.1177/00037028231176805

**Published:** 2023-05-30

**Authors:** H. Georg Schulze, Shreyas Rangan, Martha Z. Vardaki, Michael W. Blades, Robin F. B. Turner, James M. Piret

**Affiliations:** 1 Independent, Monte do Tojal, Hortinhas, Terena, Portugal; 2 Michael Smith Laboratories, 8166 The University of British Columbia, Vancouver, BC, Canada; 3 School of Biomedical Engineering, 8166 The University of British Columbia, Vancouver, BC, Canada; 4 Institute of Chemical Biology, National Hellenic Research Foundation, Athens, Greece; 5 Department of Chemistry, 8166 The University of British Columbia, Vancouver, BC, Canada; 6 Department of Electrical and Computer Engineering, 8166 The University of British Columbia, Vancouver, BC, Canada; 7 Department of Chemical and Biological Engineering, 8166 The University of British Columbia, Vancouver, BC, Canada

**Keywords:** Raman spectroscopy, peak fitting, Gaussian distributions, resolution enhancement, two-dimensional correlation spectroscopy, 2D-COS, principal component analysis, PCA, correlation structure, mammalian cells

## Abstract

Spectroscopic peak parameters are important since they provide information about the analyte under study. Besides obtaining these parameters, peak fitting also resolves overlapped peaks. Thus, the obtained parameters should permit the construction of a higher-resolution version of the original spectrum. However, peak fitting is not an easy task due to computational reasons and because the true nature of the analyte is often unknown. These difficulties are major impediments when large hyperspectral data sets need to be processed rapidly, such as for manufacturing process control. We have developed a novel and relatively fast two-part algorithm to perform peak fitting and resolution enhancement on such data sets. In the first part of the algorithm, estimates of the total number of bands and their parameters were obtained from a representative spectrum in the data set, using a combination of techniques. Starting with these parameter estimates, all the spectra were then iteratively and rapidly fitted with Gaussian bands, exploiting intrinsic features of the Gaussian distribution with vector operations. The best fits for each spectrum were retained. By reducing the obtained bandwidths and commensurately increasing their amplitudes, high-resolution spectra were constructed that greatly improved correlation-based analyses. We tested the performance of the algorithm on synthetic spectra to confirm that this method could recover the ground truth correlations between highly overlapped peaks. To assess effective peak resolution, the method was applied to low-resolution spectra of glucose and compared to results from high-resolution spectra. We then processed a larger spectral data set from mammalian cells, fixed with methanol or air drying, to demonstrate the resolution enhancement of the algorithm on complex spectra and the effects of resolution-enhanced spectra on two-dimensional correlation spectroscopy and principal component analyses. The results indicated that the algorithm would allow users to obtain high-resolution spectra relatively fast and permit the recovery of important aspects of the data's intrinsic correlation structure.

## Graphical abstract

**Graphical Abstract:**
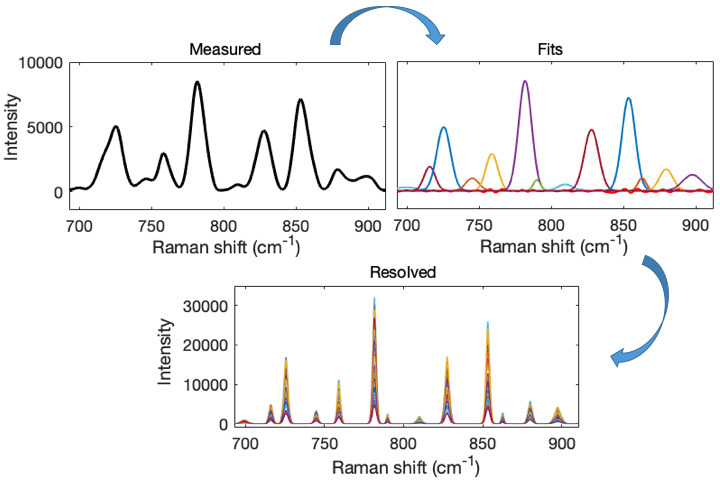
This is a visual representation of the abstract.

## Introduction

Intrinsic overlapping Raman bands in hyperspectral data sets can cause mutual interference that complicates the analysis of the spectra. These overlaps can obscure a number of “fused” bands as well as their band parameters such as amplitudes, widths, and positions. The attributes of intrinsic Lorentzian Raman bands are further distorted by spectrometer instrument point spread functions (IPSFs) that cause a “blurring”, thus additional distortion, including overlap of bands. In scanning systems, these IPSFs tend to be either Gaussian, when predominantly affected by optical elements, or triangular when dominated by slit effects.^1,2^ Therefore, correct band parameter values can be difficult to obtain, compromising the interpretation of analyte changes under study.

Principal component analysis (PCA)^3,4^ and two-dimensional correlation spectroscopy (2D-COS) and its variants,^5–9^ such as multisource correlation analysis,^10^ are dependent on correlated changes between variables. Consequently, the recovery of correct Raman band amplitudes is critically important to optimally deploy such analysis methods. For example, where two overlapping bands change in uncorrelated ways, their amplitudes are mutually modified in ways that de-correlate them from other bands with which they are truly correlated. Moreover, these amplitude modifications can give rise to complex spectral features that are difficult to interpret. Thus, methods that appropriately reduce band overlap should improve the performance of such correlation-dependent analysis methods.^11^

Diverse methods have been developed to reduce band overlap, and they generally fall into three categories: (i) band narrowing, (ii) deconvolution, and (iii) peak fitting. These have been evaluated to gain insight into their respective strengths and weaknesses.^12^ Briefly, band narrowing methods increase the separation between overlapping bands by decreasing their bandwidths, but their amplitudes are not affected by virtue of being constrained to the corresponding intensities in the original spectrum. However, no explicit estimates of the peak positions, widths, and amplitudes are obtained. Hence, both interpretation and presentation benefits result from reduced overlap, whereas no benefit from a recovered correlation structure is obtained. For deconvolution methods, IPSFs are used to deconvolve the IPSF from the measured spectra to obtain closer-to-ground truth spectra from which more accurate correlation structures can be obtained. Aside from having to measure or estimate the IPSF, bands can be missed, or artifactual bands can emerge. In principle, however, such methods could produce both reduced band overlap and improved band amplitude correlation analyses. For peak fitting methods, theoretical probability distributions, most often those approximating Voigt distributions, are fitted to the bands in a measured spectrum. Thereafter the individual bands can be artificially narrowed to the degree desired to reduce overlaps while commensurately increasing their band amplitudes. A ground truth spectrum can then be approximated from the summation of all the reduced-width bands. Consequently, in contrast to the other approaches, peak fitting yields two additional and important benefits: (i) the obtained fits yield more useful information about the analyte such as explicit band parameters and (ii) these parameters can further be used to artificially enhance the spectrum resolution by quantitatively reducing bandwidths as needed. Hence we consider peak fitting to have the most promise for resolution enhancement^12^ and it is the focus of this work. However, though this approach can in principle reduce band overlaps and improve the correlation structure between band amplitudes, it requires appropriate estimates of the initial band parameters. Most importantly, it depends on an accurate estimate of the number of bands and their positions that are present in the spectrum and this is difficult to obtain where overlapping bands are concealed such that their features cannot easily be detected with first or second derivatives.^12^ Furthermore, peak fitting approaches are time-consuming^12–14^ and often interactive, rendering them poorly suited to the automation needed when hyperspectral data sets contain thousands of spectra and/or process control requires a rapid result.

We present here a novel algorithm for adequately preprocessed Raman spectra. It first approximates a representative Raman spectrum with a sum of Gaussian distributions. Thereafter, vectors of Gaussian band parameters are calculated iteratively until a stopping criterion is met. Though Raman spectra are generally better approximated with Voigt distributions, being convolutions of Lorentzian and Gaussian distributions, Gaussian distributions are computationally less complex to implement. We first demonstrate the application of the method using synthetic spectra and then assess its performance on measured Raman spectra of glucose and mammalian (Jurkat) cells. Despite the need to approximate spectral peaks with Gaussian distributions, and the other limitations that we address, the method provided very satisfactory results.

## Materials and Methods

### Algorithm

The algorithm consisted of two parts. In the first part, a moving window peak fitting procedure was used to provide the required estimate of the number of bands present in the spectra and their estimated parameters. In part two, we used the Gaussian distribution function:

(1) $$intensities=\left(\frac{amplitudes}{sigmas\times \sqrt{2\pi }}\right)e^{-0.5{\left(\frac{x-positions}{sigmas}\right)}^{2}}$$

where **x** is a vector of selected spectral locations, **intensities** is a vector of spectral intensities, **amplitudes** is a vector of band amplitudes, **sigmas** is a vector of band standard deviations, and **positions** is a vector of band positions. By exploiting features of this distribution, we iteratively calculated band sigmas, band amplitudes, and band positions for all the spectra in the data set using the parameters estimated in the first part. The results of each iteration were then used in the next iteration until the maximum number of iterations was performed. The parameters obtained for the minimum root mean square error (RMSE) between the sum of the Gaussian distributions and the spectrum being fitted were recorded. We used Matlab R2017b (The MathWorks Inc.) to implement the algorithm, to simulate spectra, and for spectral processing and data analyses. Below, the Matlab functions are referred to with single quotes. A nominal description of the algorithm follows below, and a high-level flowchart is provided in Fig. S1 (Supplemental Material) to illustrate its basic logical structure.

#### Part One

1. The starting spectrum was used to obtain an estimate of the number of bands in the hyperspectral data set and their parameters. We used the mean spectrum of the data set as a starting spectrum because some sets can be heterogeneous and using any single spectrum might not permit all the bands to be identified.
2. The initial number of bands and their heights were estimated using the “findPeaks” function. Thereafter the “fit” function (permitting at most eight distributions to be fitted simultaneously) was used to fit Gaussian distributions in a moving window of seven bands^12^ (starting with the lowest Raman shifts). The parameters of the central band, which is band 4, were recorded and fixed before shifting the window by one band. Thus, bands at wavenumbers lower than that of the central band were not allowed to change further during the fit. Band positions were also not allowed to change from those established with “findPeaks”. Initial band amplitude fitting coefficients were determined from their spectral intensities and width coefficients were set to 1.5 times the spectral resolution of the starting spectrum. Both were allowed to vary, with the amplitude coefficient between a lower threshold (see below) and 100 times the starting spectrum maximum, and the width coefficient between 1 and twice the initial value.
3. After processing all the bands, a spectrum estimate was calculated as the sum of bands using their parameters determined with the preceding fit. Subtracting the estimate from the starting spectrum produced high intensities in the residual where hidden bands, due to overlap, could not be determined with “findPeaks”. To obtain a more accurate estimate of the positions and intensities of obscured peaks, penalization of those parts of the fit with intensities less than the starting spectrum intensities was reduced (by half) to bias the fitted spectrum from being above the starting spectrum (Fig. S2, Supplemental Material).
4. The maximum in the residual was used to define the position and height of a new band that was then added to the set of bands.^14,15^ The moving window fit was then repeated, a new residual was calculated, and a new band was added to the set. The procedure was repeated until the maximum value of the residual no longer exceeded a threshold set for the smallest peak. We set this to 0.05 or 0.001 times the maximum value of the starting spectrum as specified further below. Alternatively, it could be defined based on the noise in the starting spectrum.
5. After finding all the peaks, the fitting procedure was repeated, but with all parameters allowed to vary and no penalization applied. The amplitude and width coefficients could vary between the limits as defined above and band positions were allowed to shift by half the spectral resolution to either side of their initial estimates.
6. The final fit coefficients were converted to band parameters and then used as starting parameters in the second part of the algorithm.

#### Part Two

The procedure was initialized by setting the maximum number of iterations to be used (e.g., 50). The “peak momentum” term by which the band standard deviations and amplitudes were adjusted was set (e.g., to 0.1). The “position momentum” term by which the band positions were adjusted was set. We set this to 0.0 to produce more consistent outcomes by ignoring true position changes and by avoiding small irregularities in spectra from causing unwanted peak position shifts. We then used the band parameters obtained in Part One for the starting spectrum to process the other spectra in the set. The parameters obtained at the iteration with the minimum RMSE were recorded. The spectral resolutions of the processed spectra could be enhanced by reducing the standard deviations of the bands, with commensurate increases in their amplitudes, to the degree desired. Spectra were narrowed to 33% of the fitted widths, a value somewhat arbitrarily chosen to produce substantial narrowing but to avoid being extreme.

### Iterating

1. We first adjusted excessive sigmas and amplitudes. For all bands, we found the spectral intensities, using the current parameters, one standard deviation to the left of the band positions and one standard deviation to the right of the band positions. We calculated the band intensities from the current parameter values at these positions. If the calculated values exceeded the corresponding spectral intensities by 5% of their value, we set the excessive standard deviations to 95% of their current values. We then determined the spectral intensities at the band positions of the current band parameters. Then, we calculated, using the current parameters, the band intensities at the same positions. If the calculated values exceeded by 5% the corresponding spectral intensities, we set the excessive amplitudes to their corresponding spectral intensities. The intention here was to avoid diverging iterations caused by potentially excessive results.
2. We then calculated new sigmas. We took advantage of a simplification of Eq. 1 by setting **x** =  **positions** in Eq. 1 which leads to:
(2) $$sigmas=amplitudes/\left(\sqrt{2\pi }\;\;\times \;\;intensities\right)$$
  - We first determined the spectral intensities at the current band positions, that is, where **x** =  **positions** in Eq. 1.
  - For all other bands within 4 standard deviations of a given band, using the respective standard deviation of each 'other' band, we calculated the other band's intensity at the given band's position and subtracted that intensity from the spectral intensity determined in (a). This removed the intensities contributed by overlapping bands and provided an estimate of the intensity of only the given band.
  - Using the most recently available amplitudes and the intensities obtained in (b), we calculated the sigmas according to Eq. 2 and limited calculated values to a maximum of (3 × spectral resolution)/2 and a minimum of (spectral resolution)/2. (This minimum value can be further reduced to compensate for the use of Gaussian rather than Voigt distributions.) Thereafter, the sigmas were updated according to:
(3) $$adjusted\;\;parameters=\frac{\left(\Delta_{k+1}+\;{parameters}_{k}\right)}{1+momentum}$$
    with $\Delta_{k+1}=\;momentum\;\;\times \;\;{parameters}_{k+1}$ , **parameters**_k_ being the existing parameter values and **parameters**_k_ _+_ _1_ being the newly calculated parameter values and using the peak momentum term.
3. We then calculated the new amplitudes one standard deviation to the right of the band positions. Here we took advantage of another simplification of Eq. 1 by setting **x** = **positions** + **sigmas** in Eq. 1 which leads to:
(4) $$amplitudes=intensities\;\;\times \;\;\left(\sqrt{2\pi }\;\;\times \;\;sigmas\right)\times e^{0.5}$$
  - For all bands, we first found the spectral intensities, using the current parameters, one standard deviation to the right of the band positions, that is, where **x** = **positions** +**sigmas** in Eq. 1.
  - For all other bands within four standard deviations of the positions used in (a), that is, one standard deviation to the right of the band center and using the respective standard deviation of each “other” band, we calculated the other band's intensity at the position used in (a) and subtracted that intensity from the spectral intensity determined in (a).
  - Using the most recently available sigmas and the intensities obtained in (b), we then calculated the amplitudes according to Eq. 4 and constrained the calculated amplitudes to no less than the minimum initial parameter estimates. Thereafter, we updated the amplitudes according to Eq. 3 using the peak momentum term.
4. The new band positions were now calculated.
  - For a given band, we calculated its spectral intensity corrected for intensity contributions from overlapping bands as in Steps 2 (a) and (b).
  - For the given band and its nearest higher shift neighbor, using the most recently available parameters, we calculated the difference in band positions $\;pd_{i,i+1}$ between the current band, i, and its neighbor, i + 1:
(5) $$pd_{i,i+1}=\sqrt{-2ln\left(\left(intensities_{i}\;\;\times \;\;\left(\sqrt{2\pi }\;\;\times \;{sigmas}_{i}\right)\right)/amplitudes_{i}\right)\times \;sigmas_{i}^{2}}$$
  - Calculating the new band position as the position plus or minus the difference determined in (b). It is essential to make the correction in the proper direction. That is, if the sum of intensities of the overlapping bands to its right exceeds that to its left, the band position needs to shift left (to lower wavenumbers). When these sums are equal, no shift occurs. Otherwise, the shift is to the right (see Fig. S3, Supplemental Material). When all the new positions were calculated, we updated them according to Eq. 3, but using the position momentum term. If this momentum term is set to zero, band positions are not adjusted and remain fixed at their initial values.
5. The amplitudes were then calculated again, using the method explained in Step 3, but one standard deviation to the left of the band positions, that is, where **x** = **positions** – **sigmas** in Eq. 1.
6. The sigmas were calculated again as explained in Step 2.
7. Following Step 6, the band positions were updated as in Step 4.
8. The parameters were then sorted by band position to maintain their sequential order.
9. An estimate of the original spectrum was then obtained by summing the individual bands calculated from their current band parameters.
10. We then determined the RMSE between the estimated and original spectrum.
11. We returned to Step 1 if the iteration number was less than the specified maximum. If not, the parameters recorded for the iteration with the minimum RMSE were used as the final, best-fit estimates for a given spectrum.

After completing the fitting of one spectrum and recording its best-fit parameters, the next spectrum in the set was now processed starting with the same band estimates originally obtained in Step One.

### Synthetic Spectra

We generated seven progressively overlapped Gaussian distribution vectors until there was substantial overlap between adjacent ones, and two additional overlapping distributions for a total of nine Gaussian bands, each of 1000 channels. We then created nine successive intensity changes for each band (numbered from lower to higher channels, respectively). The intensity changes varied in different ways. Both overlaps and intensity changes allowed us to determine what distortions, if any, occurred due to overlaps and how consistently varying band amplitudes could be recovered without and with overlaps.

**Figure 1.**
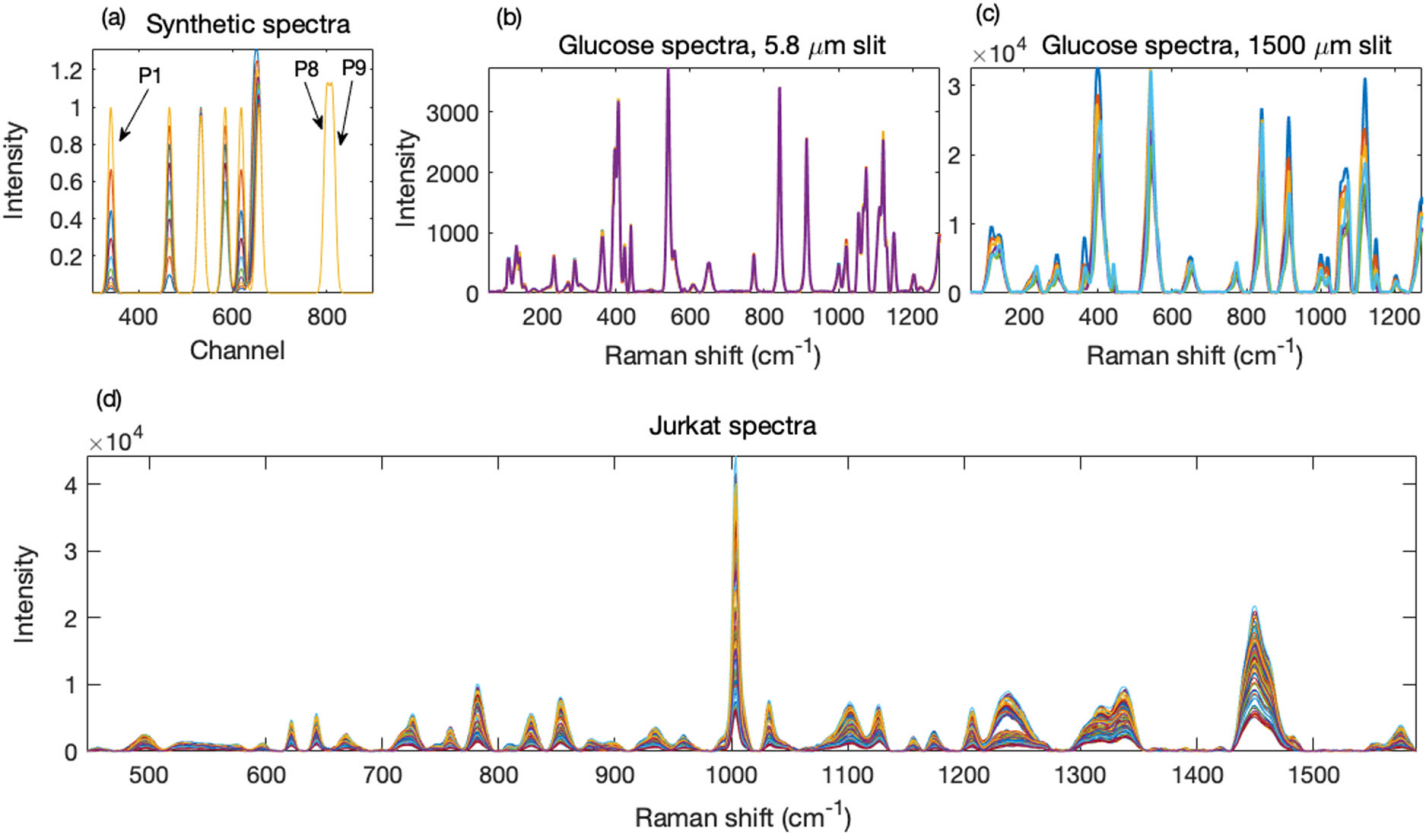
(a) Synthetic test spectra, consisting of nine bands, were generated from Gaussian distributions with varying degrees of overlap. Shown is the 300 to 900 channel region with peaks. (b, c) Glucose spectra with good and poor spectral resolutions were obtained with different spectrometer slit widths. (d) Preprocessed spectra obtained from Jurkat cells using either methanol or air drying as fixing methods.

The progressively overlapped bands were situated at channels 340, 465, 532, 584, 618, 645, and 656, and the last two overlapping bands were at 800 and 813. The band at channel 340 had a standard deviation of six channels; for all other bands, the standard deviation was three channels. All bands had a maximum of 1.00. On each change, the first band (P1) increased by 50%, starting from 0.026 of its maximum intensity. P2 increased linearly from 0.1 by 10% of its maximum. P3 decreased linearly from 1.00 to 0.95. P4 remained constant at 50% of its maximum until Step 5, from Step 6 onward it increased linearly to its maximum. P5 was identical to P1. P6 decreased linearly from its maximum to zero. It was highly overlapped with P7 such that P6 and P7 merged into one band without a shoulder. Because P7 remained constant, any distortion of its height by the rapidly changing overlapping neighbor could be more easily determined. P8 and P9 also remained constant. These intensities are provided in Table S1 (Supplemental Material). A target spectrum was produced by combining at a given stage all the bands of the ground truth set, as modified above, into a spectrum. A test spectrum was produced in the same manner, but from ground truth data with doubled standard deviations. Thus were generated 10 synthetic targets and 10 synthetic test spectra, each with nine peaks. The test spectra are shown in Fig. 1a.

### Raman Spectra

A confocal Raman microscope (InVia, Renishaw plc.) using the operating software (WiRe 4.4), a 5 × Leica objective, and excitation with a 785 nm diode laser giving ∼150 mW power (∼3 W mm^−2^) at the sample was used to collect 100 spectra from D-glucose powder (Invitrogen). An external Si standard was used for calibration. Slit widths of 1500 μm (thus limited by the ∼120 μm narrow dimension of the laser spot) and 5.8 μm, with 5 and 50 s integration time, were used to generate spectra with poor and good resolution, respectively. Their averages constituted the test and target spectra. Figures 1b and 1c show the target and test spectra, respectively.

A human T-lymphocyte cell line, Jurkat cells were inoculated into ImmunoCult-XF medium (STEMCELL Technologies) supplemented with 1 × antibiotic–antimycotic (GIBCO). After three days in culture, exponentially growing cells were either dry-fixed in saline or fixed with methanol for Raman spectroscopy. Aliquots of approximately 10^6^ cells were centrifuged at 150 g for 5 min, the supernatant was removed, and the cells were washed twice with 5 mL phosphate buffer solution. For air drying, the cell suspension was placed on a 12.5 mm diameter glass-encapsulated gold mirror (Thorlabs) and placed in a biosafety cabinet to air dry. For methanol fixing, the cells were resuspended in 100 μL of methanol and incubated at −20 °C for 20 min to fix them. The entire cell/methanol suspension was then also placed on a 12.5 mm diameter glass-encapsulated gold mirror and placed in a biosafety cabinet allowing the methanol to evaporate. Raman spectra were collected using the system described above, using a 50 × Leica objective, 10 s integration time per spectrum, and a “default” slit width of ∼50 μm.

### Data Generation and Processing

Synthetic spectra were generated as described above. Spectra were preprocessed with an automated small window moving average-based baseline-flattening method using 15 iterations,^16^ an automated coincident two-dimensional second difference cosmic ray-induced spike removal method,^17^ and smoothed with an automated peak fitting-based smoothing method.^18^ Synthetic spectra generation, preprocessing, and fitting computations were performed on a MacBook Air with a 2.2 GHz i7 processor and 8 GB 1600 MHz DDR3 memory running Apple OSX Yosemite v.10.10.5.

## Results

**Figure 2.**
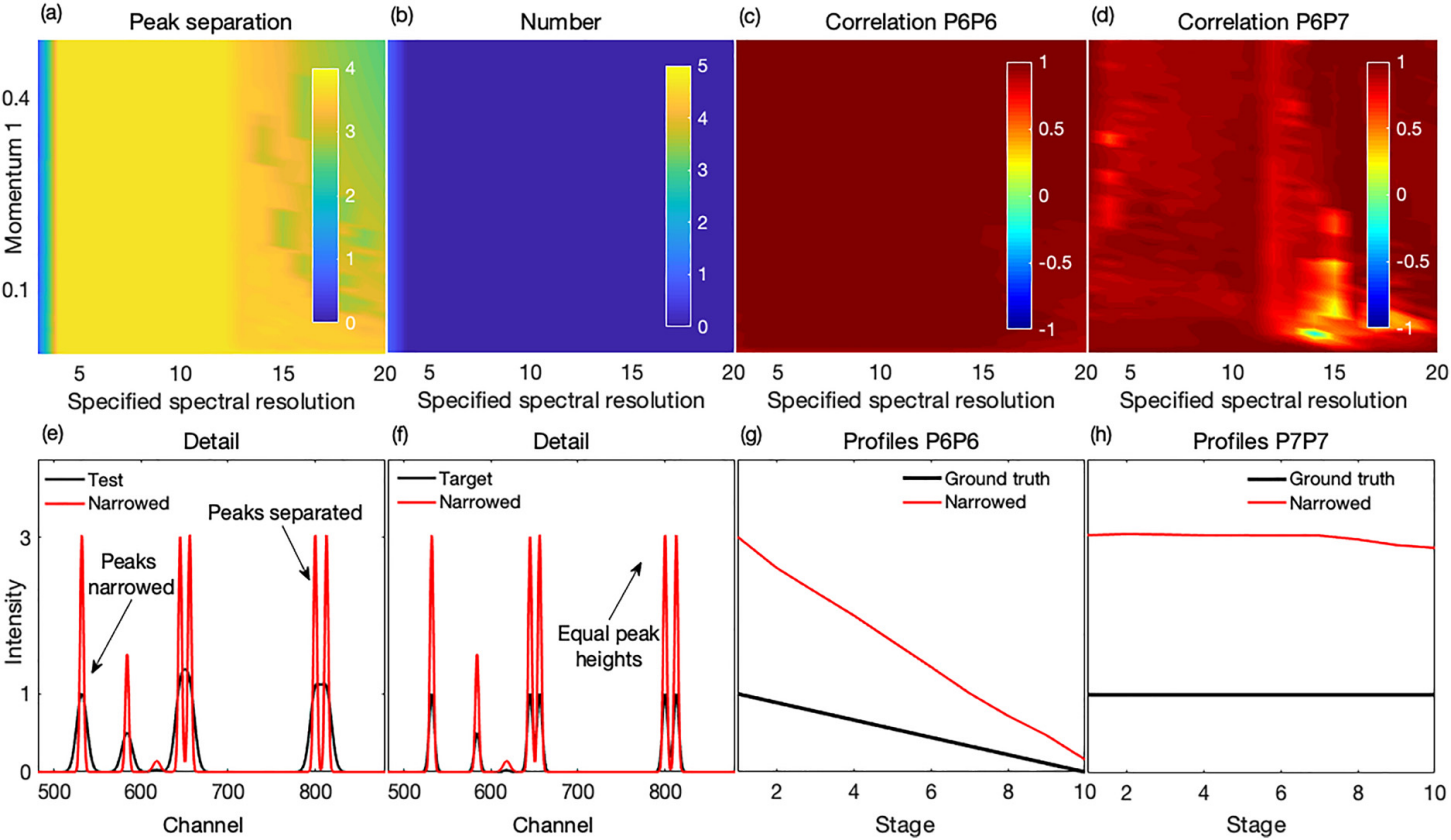
Performance of the method on synthetic spectra. (a) Peaks were mostly well separated for all specified resolutions similar to or less than that of the test spectra. (b) In general, no peaks were missed, or artifactual peaks created. (c, d) For the highly overlapped P6 and P7 with different profiles, a high correlation was observed between the narrowed recovered P6 and its corresponding target peak and a reduced correlation between the narrowed P6 and P7 suggesting a decorrelation between them. (e, f) An example narrowed spectrum, superimposed on the corresponding test and target spectra, shows effectively narrowed (with commensurately increased intensities) and separated peaks with (g, h) good recovery of their true profiles, consistent with (c, d). The example narrowed spectrum was processed with a spectral resolution setting of 12 and a peak momentum term of 0.01.

The performance of the method on the synthetic test spectra is shown in Fig. 2 and in more detail in Fig. S4 (Supplemental Material). The bands were well narrowed and hence clearly separated as shown in Figs. 2a, 2e, and 2f. Generally, all the peaks were detected, and no artifact peaks were generated, thus the number of narrowed peaks corresponded with that in the ground truth set and target spectra, see Fig. 2b and also Figs. 2e and 2f. Importantly, peak separation occurred with a recovery of the peaks’ true profiles, thus leading to a decorrelation between unrelated peaks while maintaining a correlation with their respective truth set peaks. The correlation effects are shown in Figs. 2c and 2d, and the recovered profiles in Figs. 2g and 2h. Thus, with appropriate parameter settings, an accurate performance can be obtained on noise-free synthetic spectra.

**Figure 3.**
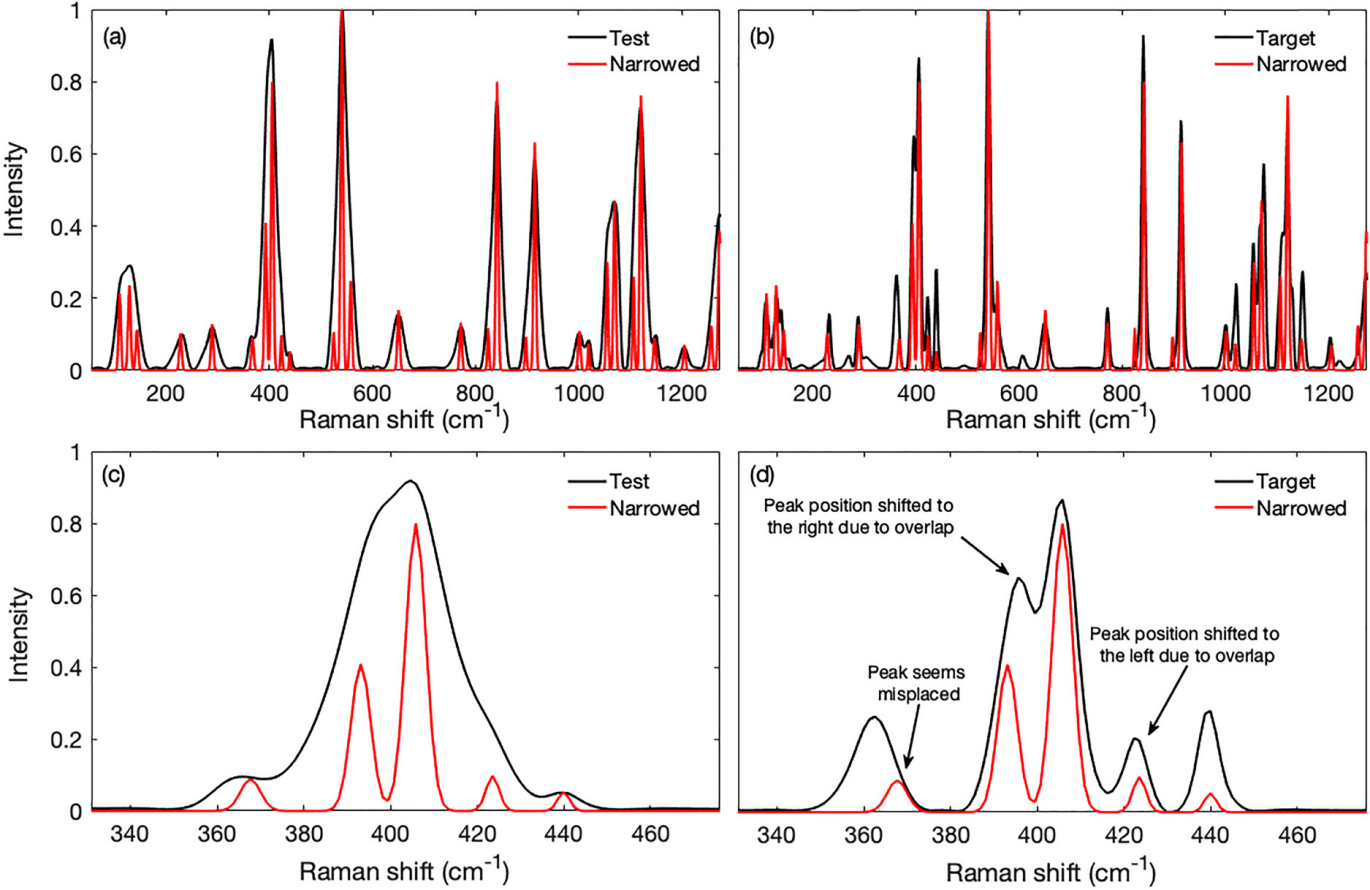
Performance of the method on real spectra obtained from powdered glucose. The test spectrum was obtained with a slit width of 1500 μm and the target spectrum with a width of 5.8 μm. The bands fitted to the test spectrum had their widths artificially narrowed to 33% of the fitted values to produce band-narrowed spectra. (a) The narrowed spectrum superimposed on that of the test spectrum, both normalized to the tallest peak near 540 cm^−1^, shows that the bands were substantially narrowed, the overlapping bands were resolved, and the hidden bands were exposed leading to improved interpretability. (b) The narrowed spectrum superimposed on that of the target spectrum shows that the narrowed bands generally corresponded with those in the high spectral resolution target spectrum. However, the peak near 365 cm^−1^ seemed displaced from its expected position. The peak heights also differed from those in the target spectrum, a discrepancy that was also visible when comparing the test and target spectra. The details can be seen in panels (c, d) where, for example, the intensity of the test spectrum peak at 440 cm^−1^ differs from the corresponding one in the target spectrum.

Figure 3 shows the results of applying the method to real spectra obtained from glucose powder. Bands were fitted to a test spectrum obtained with a slit width of 1500 μm and then narrowed to 33% of the fitted widths. We specified a spectral resolution for the test spectrum of 4 cm^−1^ (discussed below), momentum terms of 0.1, and a maximum number of iterations of 50. The band parameters corresponding to the iteration with the minimum RMSE were used to generate the final fitted and narrowed spectra. The processing time was ∼300 s. We set the minimum peak height threshold to 0.05 times the maximum peak height of the starting spectrum.

The spectrum created from the narrowed bands is superimposed on the test spectrum in Fig. 3a and on that of the high-resolution target spectrum that was obtained with a slit width of 5.8 μm in Fig. 3b. The fitting process, followed by narrowing, produced sharp peaks that were generally consistent with the high-resolution target spectrum. The resolved overlapping peaks and the overall sharpened peaks improved the presentation and interpretability of the spectrum.

A more detailed view of the cluster of bands around 400 cm^−1^ is shown in Figs. 3c and 3d. Fairly good overlaps of highly resolved peaks in the target and narrowed spectra were evident. The shifts in the narrowed overlapping peaks were expected because the apices of overlapping peaks shift toward one another as indicated in Fig. 3d, thus resolved peaks will have positions slightly away from the region of overlap (see also Fig. S3, Supplemental Material). However, peak heights differed. This might have been due to differences that existed, for unknown reasons, between the test and target spectra thus producing some ambiguity as to the proper intensity. For example, the intensity of the test spectrum peak at 440 cm^−1^ differed from the corresponding one in the target spectrum. One peak near 365 cm^−1^ also seemed misaligned, at least in the narrowed target spectrum, which was unexpected as no overlapping peaks were present in that region.

The method was also tested on a much larger number of preprocessed spectra measured from Jurkat cells and fixed within either an air-dried saline solution (50 spectra) or methanol (52 spectra). Methanol is known to leach lipids from cellular material,^19^ furthermore, the phosphatidylcholine peak near 717 cm^−1^ overlaps substantially with an adenine peak near 725 cm^−1^. Thus, we could test the performance of the method for both resolution enhancement (i.e., separating these peaks) and for profile recovery (i.e., obtaining their profiles as they should be without the distortions produced by overlap).

We constant-sum-normalized the measured spectra to give more equal prominence to the various peaks and then rescaled the normalized set to have the same maximum intensity as before normalization. The mean spectrum of this set was then used in Part One as the starting spectrum from which to obtain starting parameter values. We then proceeded in Part Two to fit all the measured spectra (not normalized) with these starting values. For processing, we specified a spectral resolution of 4 cm^−1^, a peak momentum of 0.001, and a position momentum of 0.0 to keep the results stable (more in the discussion below) and a maximum number of iterations of 15. The band parameters corresponding to the iteration with the minimum RMSE were used to generate the final fitted and narrowed spectra. Spectra were narrowed to 33% of the fitted widths. We set the minimum peak height threshold to 3 times the standard deviation of the spectrum used for starting parameters (i.e., to 187.31). Processing time was ∼250 s for Part One of the algorithm wherein 63 bands were identified and in Part Two of the algorithm it took ∼114 s to process 102 spectra (∼1.1 s per spectrum).

**Figure 4.**
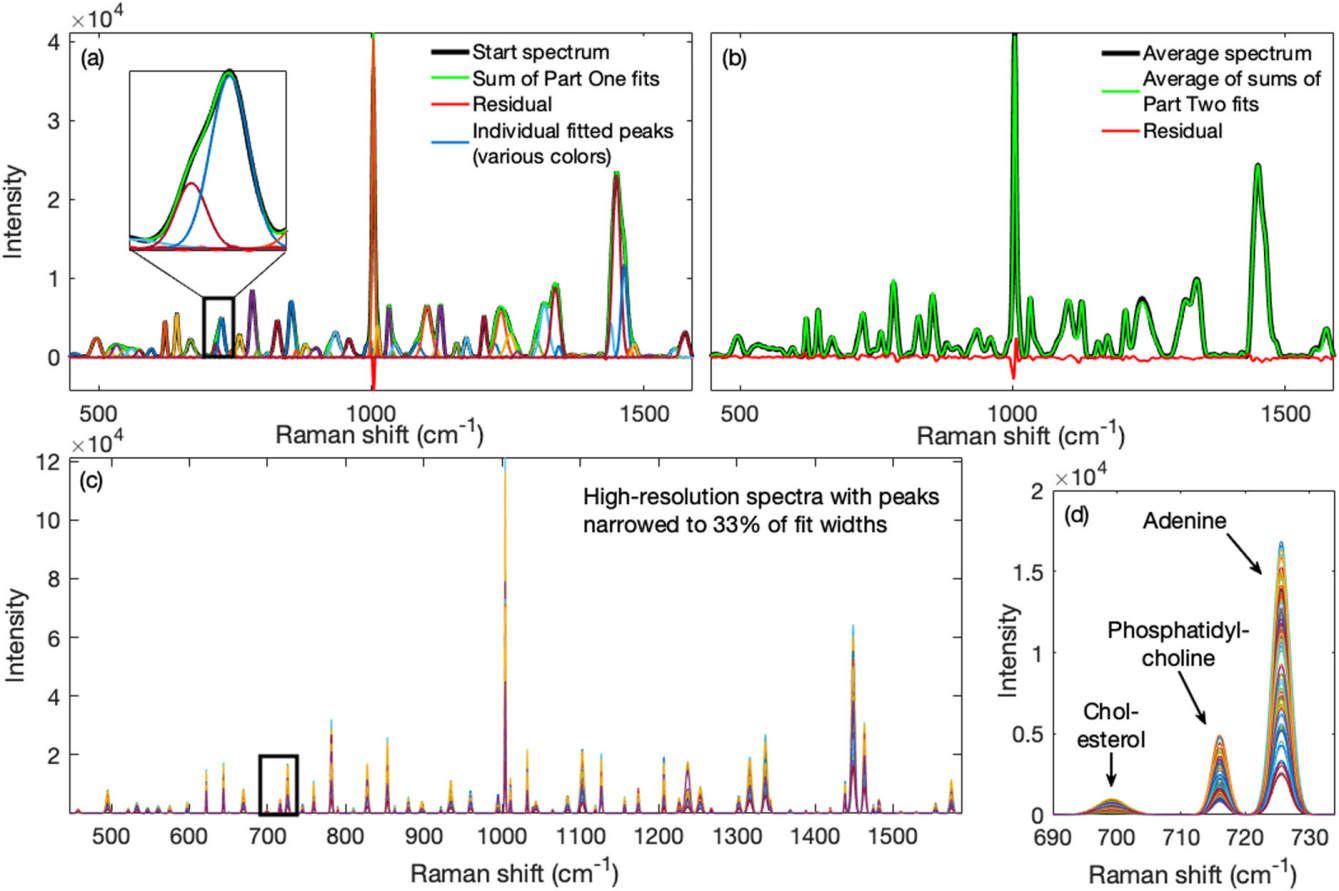
Performance of the method on 102 real spectra obtained from Jurkat cells fixed in methanol (52 spectra) or with saline air drying (50 spectra). (a) Part One: The individual fitted bands (various colors) obtained from the starting spectrum and their sum are shown superimposed on the starting spectrum; also shown is the residual between starting and fitted spectra. Inset shows fits for the overlapping phosphatidylcholine and adenine peaks. (b) Part Two: The band parameter estimates obtained in Part One were then used to process all the spectra in the set. Shown is the mean of the fitted spectra overlain on the mean spectrum in the set along with the difference between them. Using the obtained fit parameters, fitted spectra were narrowed to 33% of their final fitted widths and they are shown in (c). Details from within the black rectangle, shown in panel (d), revealed a complete separation of the phosphatidylcholine and adenine peaks after fitting and narrowing.

The results are shown in Fig. 4. The 64 individual fitted bands obtained in Part One from the starting spectrum and their sum are superimposed on the starting spectrum in Fig. 4a along with the residual between starting and fitted spectra. The sharp phenylalanine peak at ∼1003 cm^−1^ was overestimated, showing a negative feature in the residual. Though not perfect, the fit was quite good and the overlapping phosphatidylcholine peak near 717 cm^−1^ and overlapping adenine peak near 725 cm^−1^ were identified separately.

The difference between the mean measured spectrum and the mean of the fitted spectra obtained in Part Two of the algorithm, along with its residual, are shown in Fig. 4b. The mean and standard deviation of the RMSE for these spectra were 195 and 75, respectively (see also Fig. S5, Supplemental Material). In Fig. 4c are shown the narrowed spectra and the substantial resolution enhancement obtained. The narrowed spectra were reconstructed by reducing the fitted peak width parameters to 0.33 of their values and interpolating (×10) to better maintain Gaussian peak shapes after narrowing. The detail in Fig. 4d, corresponding to the black rectangle in Fig. 4c, shows that the phosphatidylcholine and overlapping adenine peaks were clearly separated.

**Figure 5.**
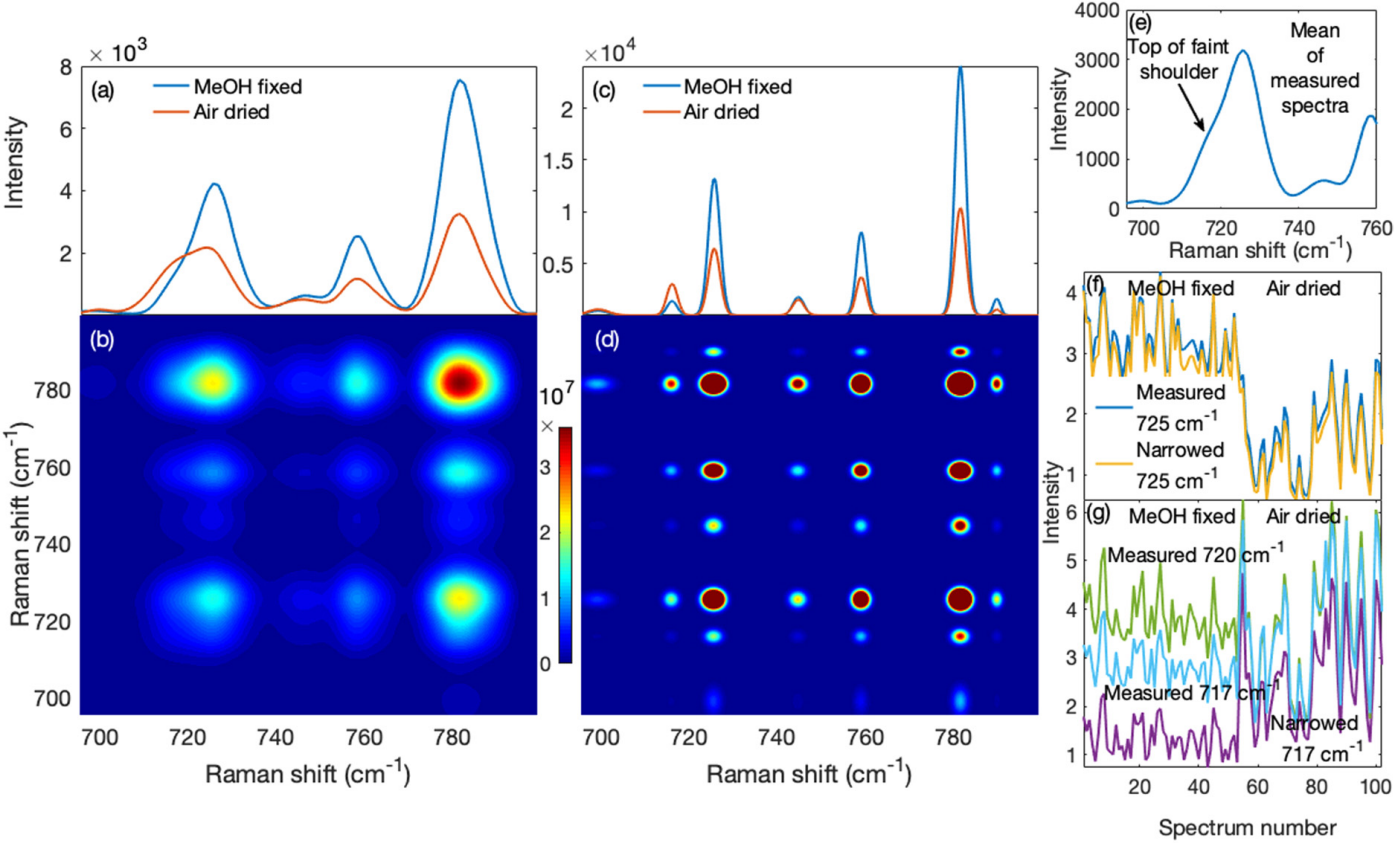
Performance of the method on 2D-COS and profile trends. (a) The mean measured spectra of methanol (MeOH) and air-dried saline (air-dried) fixed cells show that more phosphatidylcholine was retained with air-dried saline (shoulder near 717 cm^−1^) than with methanol fixing. (b) The two-dimensional correlation spectrum of the methanol fixed cells shows only auto and cross peaks for the four peaks in (a) though they have elongations that suggest the presence of hidden peaks. (c) The mean narrowed spectra of air-dried saline and methanol fixed cells show that the 717 cm^−1^ phosphatidylcholine and 717 cm^−1^ adenine peaks were resolved. Also were clearly resolved peaks near 782 cm^−1^. Consequently, (d) the two-dimensional correlation spectrum of methanol fixed cells obtained from narrowed spectra exhibits a much higher resolution than that in (b). (e) The mean spectrum of all the measured spectra shows a faint shoulder pertaining to phosphatidylcholine. (f) The standard deviation-normalized profiles of the 725 cm^−1^ adenine band across all measured and narrowed spectra were highly correlated suggesting accurate profiles in the narrowed spectra. (g) The narrowed spectra profiles of these peaks (phosphatidylcholine and adenine) were more decorrelated than their measured spectrum profiles.

The effects of the narrowed spectra on correlation-based methods such as 2D-COS are shown in Fig. 5. The mean measured spectra of the cells fixed with methanol and air-dried saline are shown in Fig. 5a. It can be seen that the peak at 725 cm^−1^ has a prominent shoulder at 717 cm^−1^ in the air-dried saline, but not the methanol fixed sample due to the loss of lipids that occur under methanol fixation. In Fig. 5b, the two-dimensional correlation spectrum for the methanol fixed measured spectra is presented. The adenine peak at 725 cm^−1^ did not show evidence of the overlapped adjacent phosphatidylcholine peak save for mild elongations in the direction of the overlap. We show the mean narrowed spectra of the cells fixed with methanol and air drying in Fig. 5c. Compared to Fig. 5a, not only was the phosphatidylcholine peak separated from the overlapping adenine peak, the nucleic acid peak at ∼780 cm^−1^ was also revealed to have consisted of overlapping peaks. This produced, for the methanol fixed narrowed spectra, a two-dimensional correlation spectrum in Fig. 5d of a much higher resolution than in Fig. 5b.

In Fig. 5e, we show the mean of the measured spectra for the entire set (both methanol and air-dried saline). The 725 cm^−1^ adenine peak has a faint shoulder on the lower shift side and, suspecting a hidden peak, one might choose to examine the adenine peak's profile or trend using the wavenumber corresponding to the top of the shoulder as representative of this peak. However, this profile is still influenced by the taller overlapping 725 cm^−1^ adenine peak as we describe below. In Fig. 5f, we show that the 725 cm^−1^ adenine profile of the measured spectra, normalized by its standard deviation, was nearly identical to that of the narrowed spectra (correlation coefficient *r*  =  0.9986; *P* < 0.0001). This argues against systematic or random variations introduced by the algorithm, consistent with the results shown in Figs. 2g and 2h. Thus, the substantial difference for the narrowed spectra between the phosphatidylcholine profile at 717 cm^−1^, shown in Fig. 5g, and the 725 cm^−1^ adenine profile in Fig. 5f, reveals that they were decorrelated (*r*  =  −0.1798; *P*  =  0.0706). In contrast, the profiles for the unresolved measured spectra at 717 and 720 cm^−1^ (where one might choose the profile) were more correlated with the 725 cm^−1^ profile (*r*  =  0.1408; *P*  =  0.1580 and *r*  =  0.5619; *P* < 0.0001, respectively).

**Figure 6.**
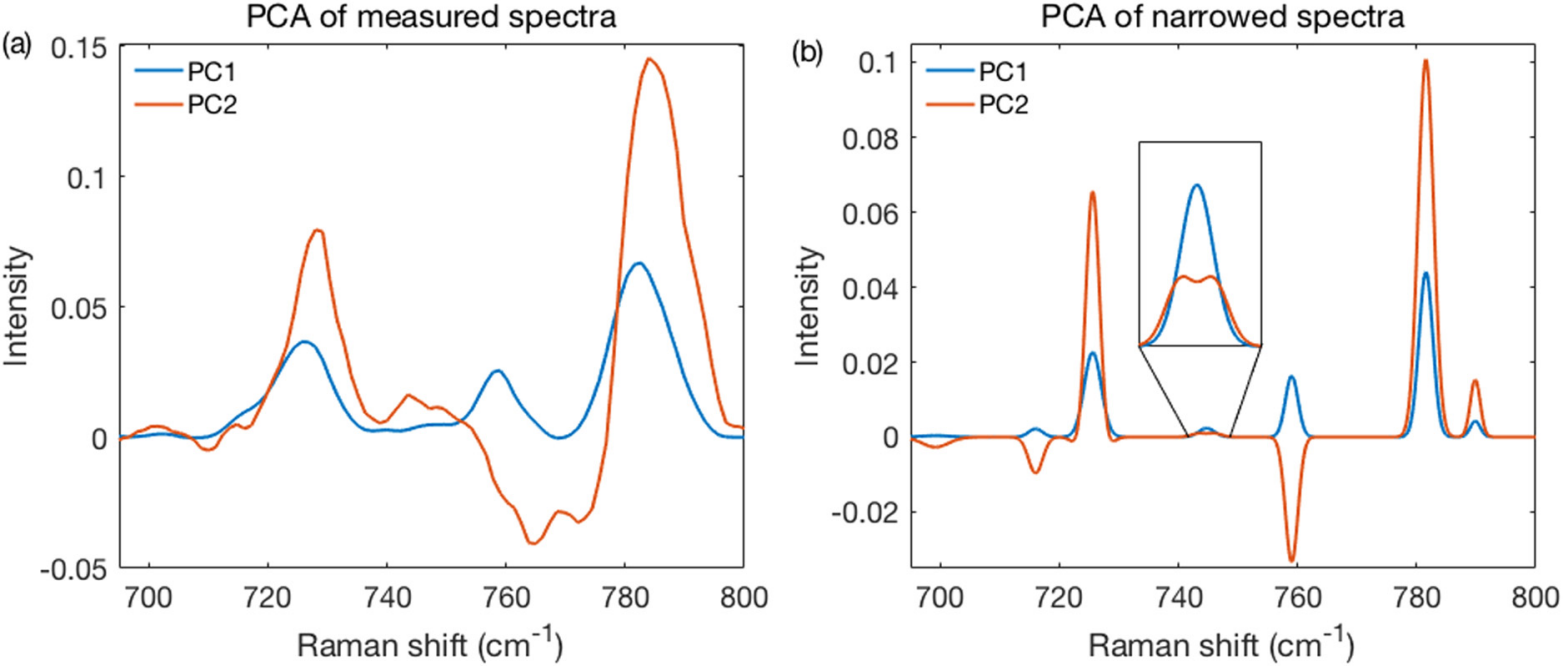
Performance of the method using PCA. (a) The PC1 and PC2 loadings of fixed measured spectra. (b) Same as in (a), but for narrowed spectra. The sharper peaks produced loadings far more clearly related to specific peaks. Inset provides details of a 746 cm^−1^ peak.

The high resolution obtained with narrowed spectra also leads to a substantial resolution improvement when using PCA. The loadings on the first principal component (PC1) and second principal component (PC2) from the methanol fixed measured and narrowed spectra are shown in Figs. 6a and 6b. Besides a higher resolution, the loadings appear easier to interpret because fewer peaks overlap and are therefore not distorted by unrelated changes. For example, the PC2 in Fig. 6b shows correlated changes between peaks at 725, 746, 781.5, and 789 cm^−1^, all assigned to nucleic acids.^20^ We have also observed, in some loadings, that peaks can exhibit features reminiscent of second derivatives. An incipient one (not yet very second derivative-like) is present in the Fig. 6b inset. These effects seem to have originated from peak broadening, rather than peak shifts as the latter were not permitted (see Fig. S6, Supplemental Material). Thus, the higher resolution uncovered further, more subtle, correlation-related effects that need to be examined in future work. Specifically, it needs to be understood to what extent these might be artifacts related to the use of preprocessing and fitting algorithms or rather reflect real aspects of the samples analyzed.

## Discussion

The growth in applications that benefit from hyperspectral data analysis has in turn resulted in a growing interest in fully automated spectral preprocessing methods.^14,16–18,21–24^ However, we have not been able to accomplish this with the algorithm presented here. It is, at best, semiautomated. The difficulties pertain to selecting effective parameter settings for the algorithm when processing measured spectra. Thus, the spectral resolution must be determined or specified, and we found that a better result is obtained by selecting a value smaller than the actual spectral resolution. This appears to avoid, to some extent, degenerate solutions where overlapping peaks in the starting spectrum can be fitted equally well with one broad peak or two narrower ones. A minimum peak threshold also must be specified, though this can be done in a principled manner, for example, by specifying the threshold in terms of the average level of noise in the spectra being processed. Furthermore, the threshold affects the number of peaks identified and this has a bearing on the ultimate resolution and peak profiles obtained. The example of the overlapping phosphatidylcholine and adenine peaks is relevant here (Fig. 5): if the threshold were set too high to permit identification of the weaker phosphatidylcholine peak, the peaks would not have been resolved and the adenine peak profile might have been distorted by the presence of the unresolved phosphatidylcholine peak. Momentum terms must be specified, and appropriate values need to be determined with a trial-and-error approach, perhaps using a small subset of the data. Setting the position momentum term to zero avoids small shifts in the peaks due to residual imperfections in the spectra that tend to blur the peaks and thus broaden them in the aggregate again. However, this also prevents the detection of real shifts when they occur in the data set. Thus, users might have to decide whether to rather set this term to a small value to achieve the proper trade-offs given their data. The maximum number of iterations has to be specified such that the minimum RMSE occurs at an iteration within this maximum. Furthermore, spectra must be baseline-corrected, have cosmic ray-induced spikes removed, and be smoothed before peak fitting. The spectra do not need to be normalized as the resolution-enhanced spectra might offer more accurate normalization.

Chen and Garland^13^ devised a method to address automation by fitting Pearson VII distributions to large sets of infrared absorption spectra. To estimate peak parameters, a second derivative approach determined peak positions, peak widths were estimated based on the system under study, shapes were assessed to be near Lorentzian and amplitudes were algebraically solved given initial values for the other parameters. The method then optimizes peak parameters from these starting parameters using the simplex optimization method. Appropriate starting parameters need to be determined from a chosen spectrum in the data set with sufficient information to provide adequate estimates. Optimized parameters for this spectrum then serve as starting estimates for the adjacent spectrum and the latter's optimized parameters as starting values for the next spectrum and so forth to fit the entire array of spectra.

In contrast, our method uses Gaussian distributions and algebraically solves iteratively for all the parameters given the initial values. To obtain starting parameters, in Part One of the algorithm we used the mean spectrum of the data set to 'amalgamate' all the peaks in the data set into one spectrum from which they could be recovered. However, obtaining the mean of the spectra in this way diminishes the contribution of small peaks that occur infrequently but that might still be of interest or importance. To overcome this problem, we normalized all the spectra to a constant sum and scaled the results as described earlier. Estimates of peak parameters were determined using a combination of derivatives, peak fitting, and peak subtraction. An alternative approach to ameliorate this effect might be to use *k*-means or *k*-medoids clustering to partition the data set into a number of clusters of similar spectra, pick the centroids or medoids of these clusters as starting spectra to find the number of bands and their parameter estimates for each cluster, and then combine the results before processing the spectra in Part Two of the algorithm.

Though the performance of the method has been demonstrated in detail above, another critical issue needs to be addressed. Fitting methods are often used interactively, and where automated, can be time-consuming due to computational complexity. Part One is the most time-consuming segment of the algorithm as it took about 250 s to obtain parameter estimates from a starting spectrum of 50 to 80 peaks. In Part Two, fitting proceeds much faster and it took about 1.1 s per spectrum. However, this is mostly dependent on the number of iterations specified and the choice of the peak momentum term, such that smaller terms can increase processing times. Nevertheless, our processing times demonstrate the feasibility of fitting and narrowing large numbers of spectra in a semiautomated manner, once appropriate parameter settings have been established.

First, it is important to add comments about the specific limitations of the algorithm used in this work. First, it is our view that finding and approximately locating all the peaks in a spectrum is the most pressing problem that would benefit from continued attention. With real spectra, where irregularities might remain even after smoothing, fitting might produce chemically meaningless phantom peaks, particularly where spectra have a poor signal-to-noise ratio. In addition, where peaks are often highly overlapped, the use of derivatives to locate real peaks will also have a limited utility such that other methods, or combinations of methods, should be considered. Our approach, as demonstrated in Fig. S2 (Supplemental Material), was to use a moving window^12^ combination of slightly constrained fitting with subtraction to locate real peaks and reduce the risk of creating phantom peaks. This was followed by unconstrained fitting of all the peaks to improve their parameter estimates. However, as the sole purpose of Part One of the algorithm is to determine the correct number of peaks in a spectrum and their approximate locations, it might be dispensed with if a better technique is available. Ideally, this might be incorporated in Part Two of the algorithm. Missing values can be inferred from data using an alternating least squares approach,^25^ thus Part Two of the algorithm might be adapted to deal with missing peaks and their parameters, though some limit might exist to the proportion of missing values that can be handled.

Second, the algorithm also fits Gaussian bands to Raman peaks that are Voigt distributions, though they have varying degrees of Gaussian character, thus there is an inherent drawback that might be overcome by extending the method to Voigt distributions. Voigt distributions are mathematically complicated and time-consuming to optimize though Chen and Dai^26^ have developed an accelerated Levenberg optimization method for the automated fitting of individual Voigt distributions to Raman spectra. However, they might be difficult to implement in the manner that we pursued here. We have not investigated an extension to Voigt distributions, Pearson VII, or other suitable approximations.

## Conclusion

The novel two-part algorithm described here for performing peak fitting and resolution enhancement on large numbers of spectra was tested on both synthetic and measured spectra. Estimates of the total number of bands and their parameters were obtained in Part One of the algorithm from a suitable starting spectrum in, or derived from, the hyperspectral data set. In Part Two, these parameter estimates served as starting values to rapidly fit Gaussian bands, iteratively, to all the spectra using a vector-based approach. The best fits for each spectrum were retained. Bandwidths could be reduced, and their amplitudes commensurately increased to produce high-resolution spectra from these band parameters. We demonstrated with synthetic spectra that the intrinsic correlation structure of the data can be recovered in the obtained high-resolution spectra. We then applied the method to real spectra showing that the obtained high-resolution spectra greatly improved correlation-based analyses such as 2D-COS and PCA. Consequently, we believe that our method will allow users to obtain high-resolution spectra with good intrinsic correlation structure fidelity and with processing times fast enough to make feasible the processing of large hyperspectral data sets.

## Supplemental Material

sj-pdf-1-asp-10.1177_00037028231176805 - Supplemental material for Rapid Vector-Based Peak Fitting and Resolution Enhancement for Correlation Analyses of Raman Hyperspectra Click here for additional data file. Supplemental material, sj-pdf-1-asp-10.1177_00037028231176805 for Rapid Vector-Based Peak Fitting and Resolution Enhancement for Correlation Analyses of Raman Hyperspectra by H. Georg Schulze, Shreyas Rangan, Martha Z. Vardaki, Michael W. Blades and Robin F. B. Turner, James M. Piret in Applied Spectroscopy
