# Supplementary material for: Rapid Vector-Based Peak Fitting and Resolution Enhancement for Correlation Analyses of Raman Hyperspectra
Source: Appl Spectrosc. 2023 May 30;77(8):957–69. doi: 10.1177/00037028231176805 (PMC10543951; doi:10.1177/00037028231176805)
Supplement: sj-pdf-1-asp-10.1177_00037028231176805 - Supplemental material for Rapid Vector-Based Peak Fitting and Resolution Enhancement for Correlation Analyses of Raman Hyperspectra [file sj-pdf-1-asp-10.1177_00037028231176805.pdf]

Rapid Vector-Based Peak Fitting and Resolution Enhancement for Correlation Analyses of Raman Hyperspectra

Supporting Material

H. Georg Schulze,[1] Shreyas Rangan,[2,3] Martha Z. Vardaki,[4] Michael W. Blades[5],

Robin F. B. Turner,[2,5,6*] and James M. Piret,[2,3,7*]

1. Independent, Monte do Tojal, Caixa Postal 128, Hortinhas, Terena, Portugal, 7250-069.
2. Michael Smith Laboratories, The University of British Columbia, 2185 East Mall, Vancouver, BC, Canada, V6T 1Z4.
3. School of Biomedical Engineering, The University of British Columbia, 2222 Health Sciences Mall, Vancouver, BC, Canada, V6T 1Z3.
4. Institute of Chemical Biology, National Hellenic Research Foundation, 48 Vassileos Constantinou Avenue, Athens, 11635, Greece.
5. Department of Chemistry, The University of British Columbia, 2036 Main Mall, Vancouver, BC, Canada, V6T 1Z1.
6. Department of Electrical and Computer Engineering, The University of British Columbia, 2332 Main Mall, Vancouver, BC, Canada, V6T 1Z4.
7. Department of Chemical and Biological Engineering, The University of British Columbia, 2360 East Mall, Vancouver, BC, Canada, V6T 1Z3.

[*]Corresponding Authors (*turner@msl.ubc.ca* and *james.piret@ubc.ca*)

Here we present Table S1 with the intensities of the synthetic data peaks. We also present figures to illustrate (i) a high-level flow chart (Fig. S1), (ii) the effects of applying a bias to the moving window peak fitting procedure (Fig. S2), (iii) the direction in which to adjust peak positions (Fig. S3), (iv) all the figures of merit determined for the synthetic spectra (Fig. S4) and (v) all the fitted Jurkat cell spectra with their residuals (Fig. S5).

Table S1

Intensity changes for the progressively overlapping synthetic peaks. Shown are ground truth intensities, thus without the effects of overlaps.

P1	0,03	0,04	0,06	0,09	0,13	0,20	0,30	0,44	0,67	1,00
P2	0,10	0,20	0,30	0,40	0,50	0,60	0,70	0,80	0,90	1,00
P3	1,00	0,99	0,99	0,98	0,98	0,97	0,97	0,96	0,96	0,95
P4	0,50	0,50	0,50	0,50	0,50	0,60	0,70	0,80	0,90	1,00
P5	0,03	0,04	0,06	0,09	0,13	0,20	0,30	0,44	0,67	1,00
P6	1,00	0,89	0,78	0,67	0,56	0,44	0,33	0,22	0,11	0,00
P7	1,00	1,00	1,00	1,00	1,00	1,00	1,00	1,00	1,00	1,00
P8	1,00	1,00	1,00	1,00	1,00	1,00	1,00	1,00	1,00	1,00
P9	1,00	1,00	1,00	1,00	1,00	1,00	1,00	1,00	1,00	1,00

Figure S1 (Below)

Simplified high-level flow chart illustrating the algorithmic logic. The iteration steps of Part Two of the algorithm are shown by the numbers on the left. A detailed description of the algorithm is provided in the main text.

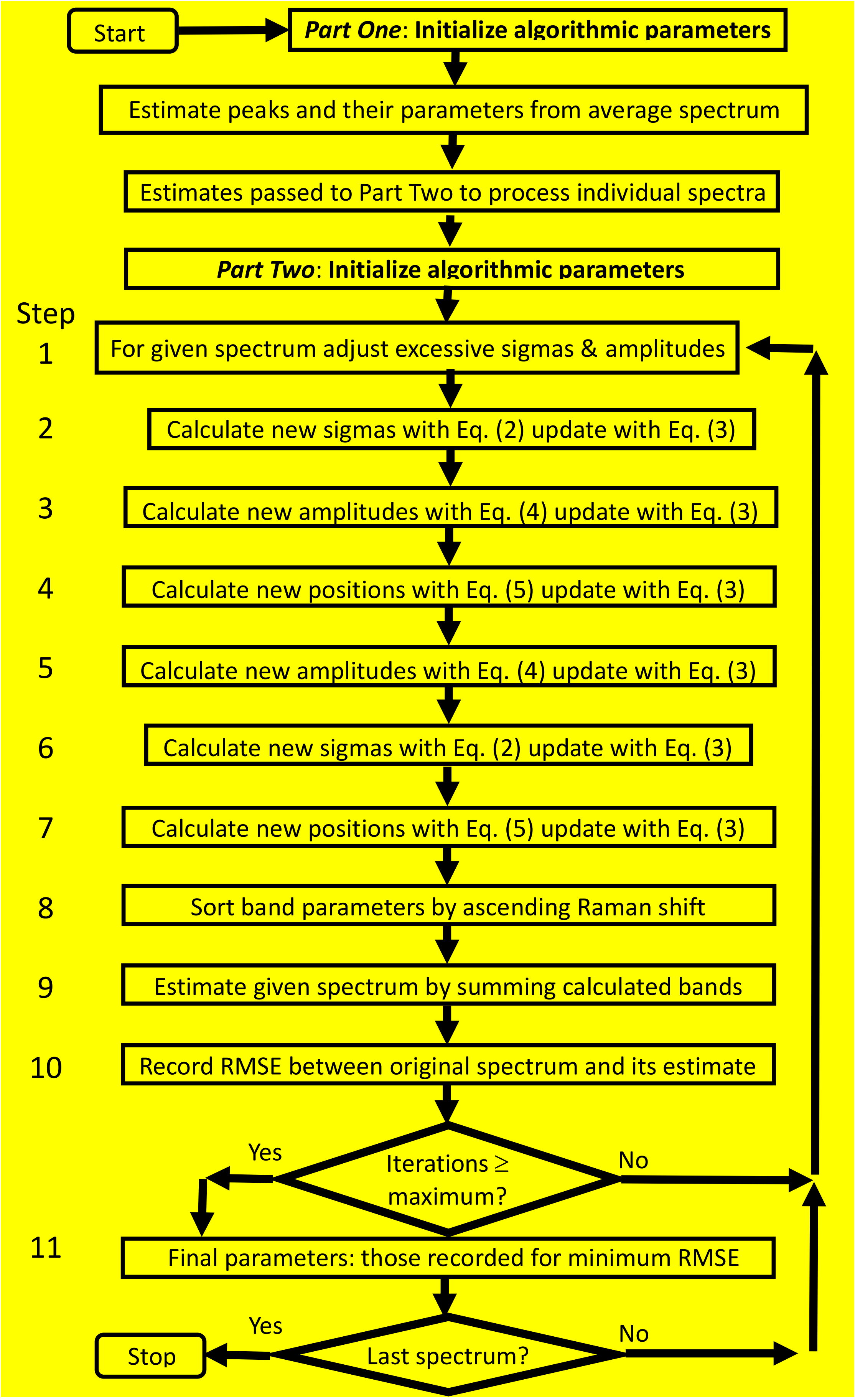
Start
Part One: Initialize algorithmic parameters
Estimate peaks and their parameters from average spectrum
Estimates passed to Part Two to process individual spectra
Part Two: Initialize algorithmic parameters
Step
1
For given spectrum adjust excessive sigmas & amplitudes
2
Calculate new sigmas with Eq. (2) update with Eq. (3)
3
Calculate new amplitudes with Eq. (4) update with Eq. (3)
4
Calculate new positions with Eq. (5) update with Eq. (3)
5
Calculate new amplitudes with Eq. (4) update with Eq. (3)
6
Calculate new sigmas with Eq. (2) update with Eq. (3)
7
Calculate new positions with Eq. (5) update with Eq. (3)
8
Sort band parameters by ascending Raman shift
9
Estimate given spectrum by summing calculated bands
10
Record RMSE between original spectrum and its estimate
Yes
Iterations ≥ maximum?
No
11
Final parameters: those recorded for minimum RMSE
Yes
Stop
Last spectrum?
No

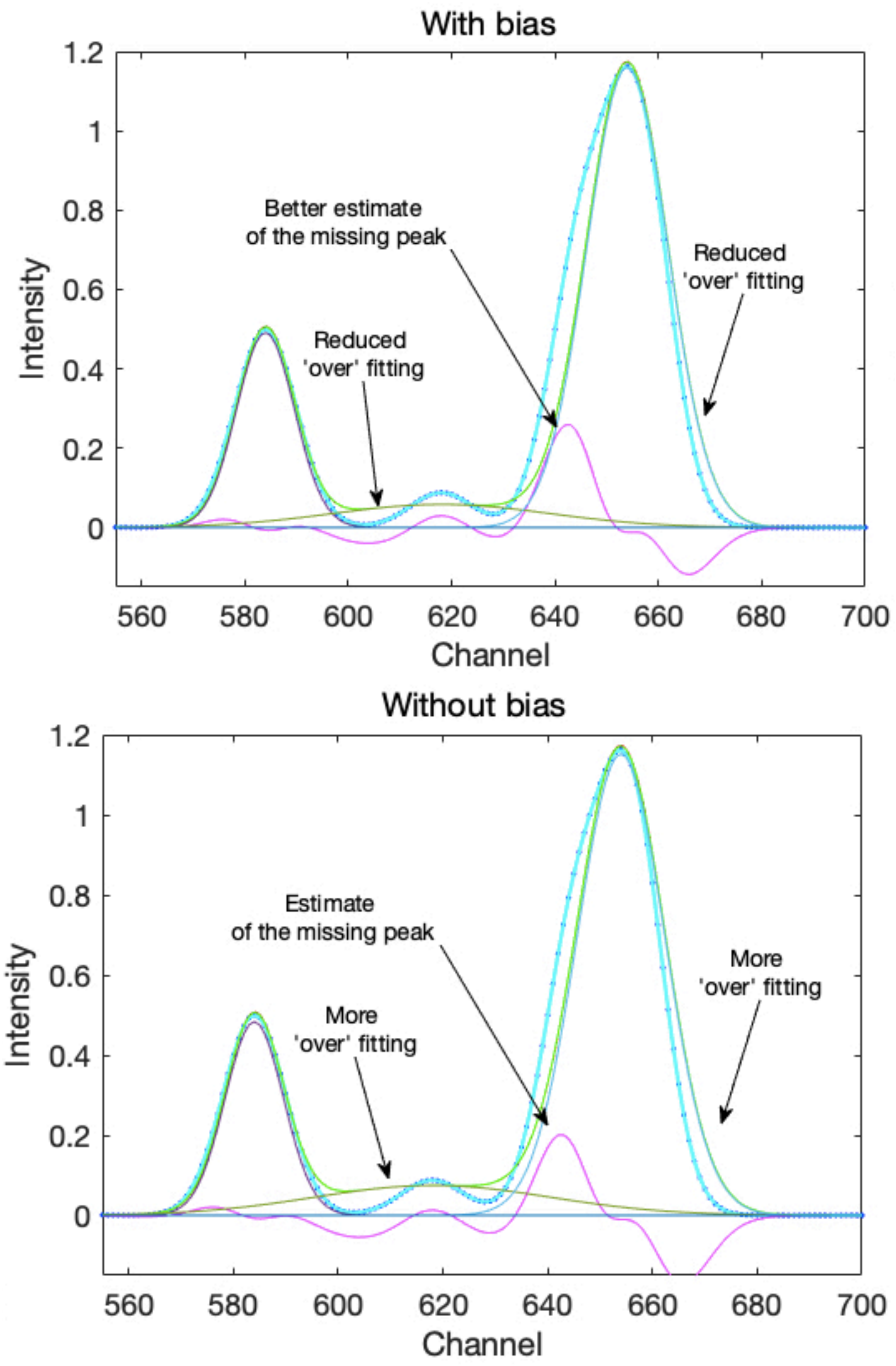

Figure S2

The top panel shows the result of moving window curve fitting with bias to promote fitting under or at the spectrum and the bottom panel the result of fitting without the bias. Fitting with the bias tends to produce a better estimate of the location and intensity of a missing peak.

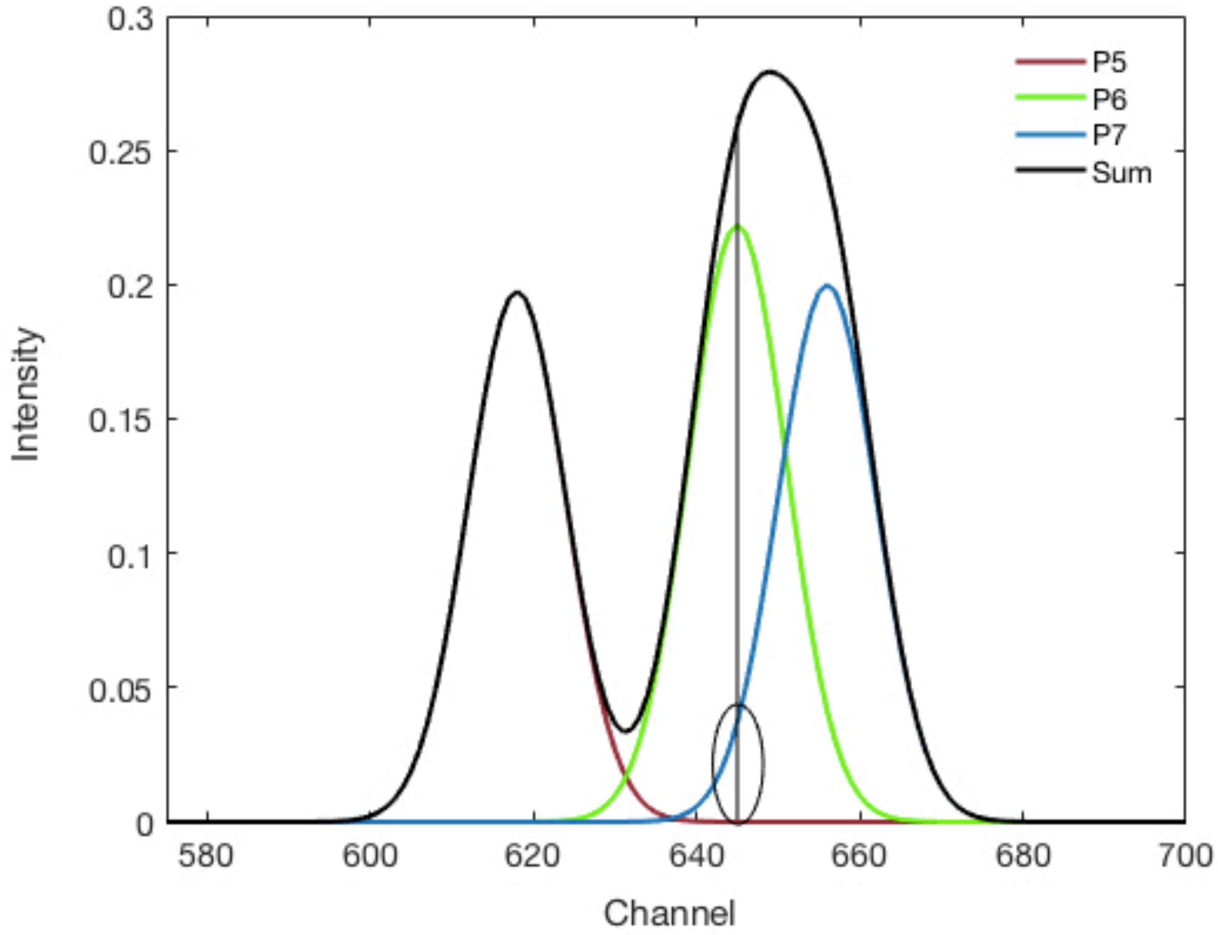

Figure S3

The figure shows that P5 has negligible overlap with P6 while P7 has substantial overlap with P6 (oval) causing the apex of the sum of bands to shift away from the apex of P6 (vertical line). Thus, the shift is in the direction of the overlapping band with the most intensity at the position of the band (P6) under consideration. Therefore, band position adjustments must be made in the opposite direction. Where there are several overlapping bands, the argument applies to the direction with the greatest sum of intensities of the overlapping bands at the position of the band under consideration. That is, the sum of intensities of overlapping bands on the left or the sum of intensities of overlapping bands to the right of the band under consideration.

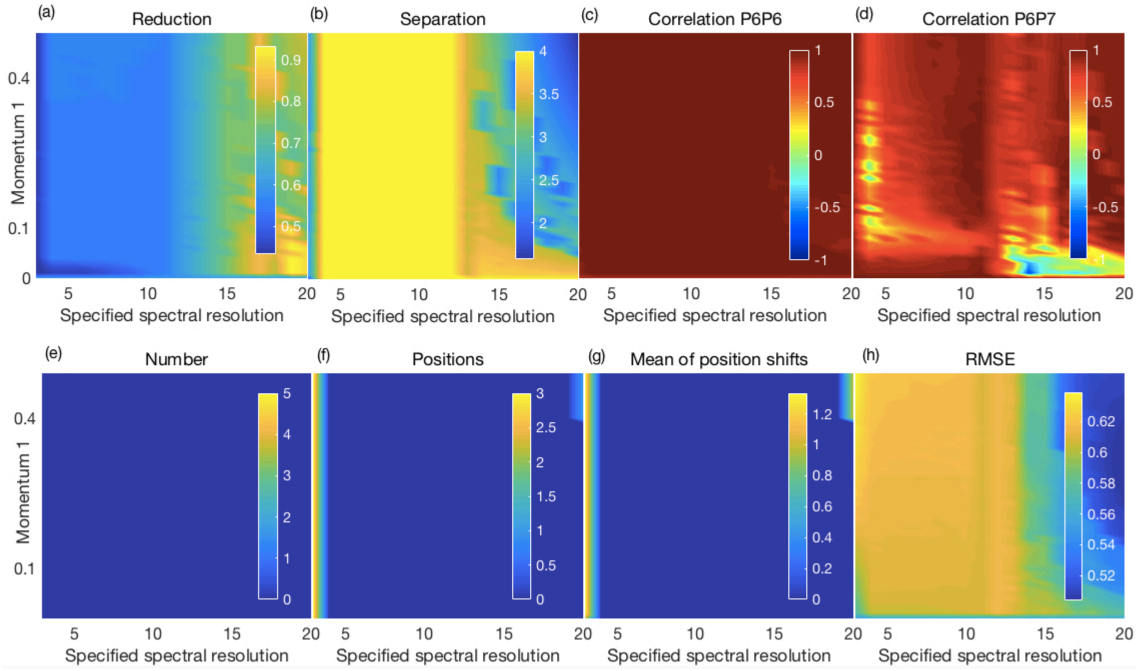

Figure S4

Performance of the method on synthetic spectra. (a) Reduction in peak width with smaller numbers indicating larger reductions. Peak reductions, relative to the test spectra, were greater for peaks with already small widths due to smaller spectral resolutions being specified. (b) Peaks were well separated for all specified resolutions similar to or less than that of the test spectra. (c, d) For the highly overlapped P6 and P7 with different profiles, a high correlation was observed between the narrowed recovered P6 and its corresponding target peak and a reduced correlation between the narrowed P6 and P7 suggesting a decorrelation between them. (e) No peaks were missed or artefactual peaks created and (f, g) all peaks were correctly located. (h) Because peak widths were artificially reduced, greater RMSEs will occur for peaks with reductions to widths less than those of the target spectra.

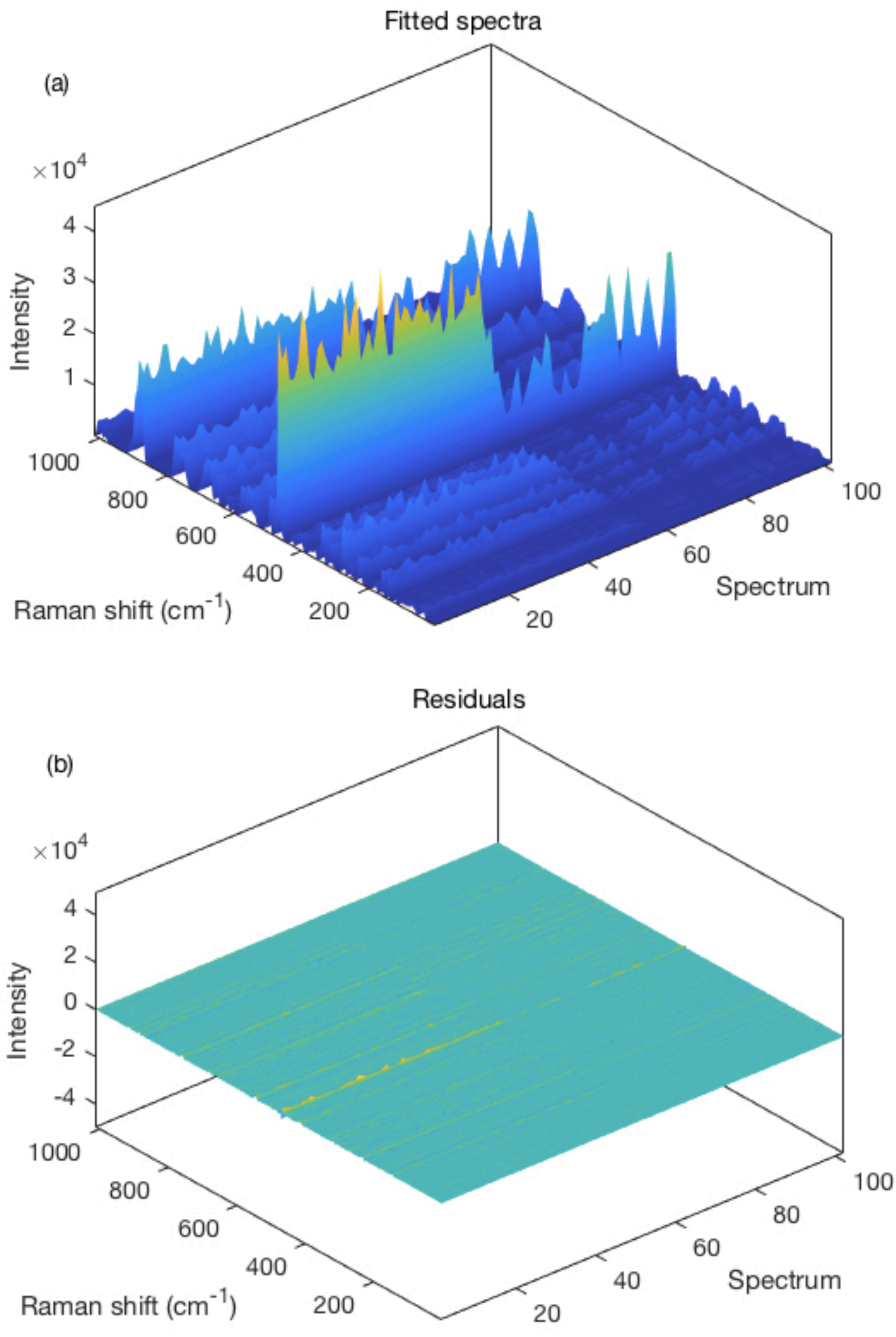

Figure S5

(a) The methanol-fixed (1 to 50) and air-dried (51 to 102) Jurkat cell spectra reconstituted from the bands fitted to the originally measured and preprocessed spectra using the obtained best fit parameters. (b) The residuals between the originally measured and preprocessed spectra and the reconstituted spectra in (a) show very small residuals with the largest deviations occurring for the 1003 cm^{-1} phenylalanine peak. This was possibly due, or partly due, to having approximated a peak with substantial Lorentzian character using a Gaussian distribution.

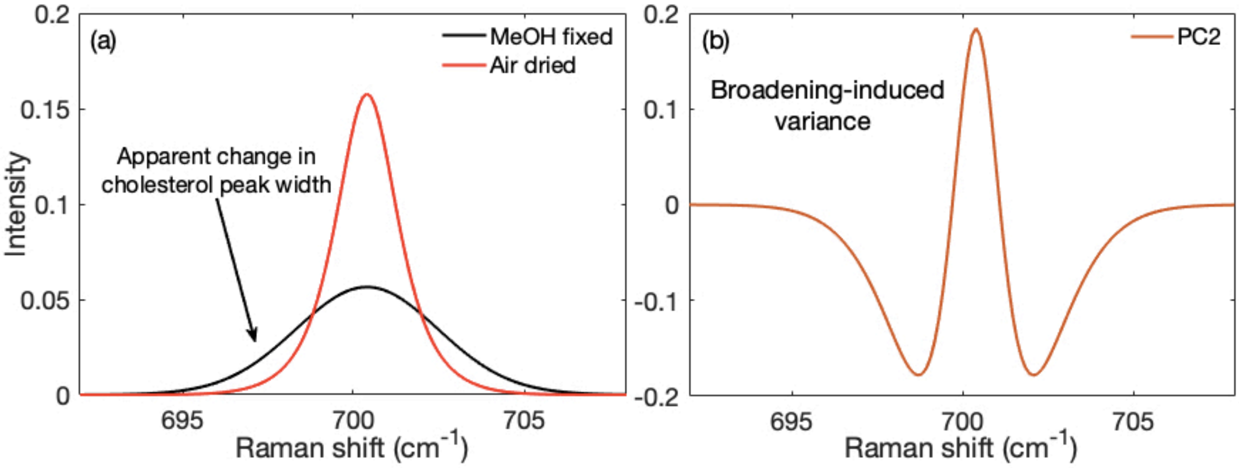

Figure S6
(a) Peak broadening in resolution-enhanced spectra produce (b) second-derivative like features in principal component loadings.
